# Artificial Intelligence Versus Human Interpretation in Diagnostic Radiology: A Systematic Review of Diagnostic Accuracy and Operational Efficiency

**DOI:** 10.7759/cureus.118954

**Published:** 2026-11-01

**Authors:** Santiago Diaz-Perdomo, María J Losada-Peña, Viviana Gutiérrez-Riobamba, Luisa F Cobaleda-Ramos, Yolercy Vasquez, Jose D Charry

**Affiliations:** 1 Medicine, Navarra University Foundation (UNINAVARRA), Neiva, COL; 2 Medicine, Faculty of Health Sciences, Surcolombiana University, Neiva, COL; 3 Research and Innovation Center (CIINA), Navarra University Foundation (UNINAVARRA), Neiva, COL

**Keywords:** artificial intelligence (ai), diagnostic radiology education, diagnostic test accuracy, operational efficiency, systematic literature review

## Abstract

Rising imaging workload and a shortage of radiology specialists have driven interest in artificial intelligence (AI) to improve diagnostic speed and accuracy. This systematic review, without meta-analysis, describes how AI affects diagnostic accuracy and operational efficiency compared with human interpretation in diagnostic radiology. Following Preferred Reporting Items for Systematic Reviews and Meta-Analyses (PRISMA 2020) and Cochrane guidance, PubMed, ClinicalKey, ScienceDirect, and Google Scholar were searched on 11 April 2026 for studies published from January 2019 onwards. Two reviewers independently performed study selection, data extraction, and a Quality Assessment of Diagnostic Accuracy Studies 2 (QUADAS-2)-based risk-of-bias assessment. Twenty-one studies were included: 11 compared standalone AI with human readers, six compared AI-assisted with unassisted readers, and four reported both; 18 evaluated task-specific image-analysis AI and three evaluated generative AI models. Because no subgroup was homogeneous enough to pool, we synthesised results narratively and stratified them by comparison type, AI category, imaging modality, and study quality. Standalone image-analysis AI achieved high sensitivity for fractures and selected chest findings but performed less well on subtle findings and lower-quality images, and in one computed tomography-referenced comparison a radiologist outperformed AI. AI assistance improved reader performance more consistently, particularly among less experienced readers. Generative models did not reach specialist performance on complex tasks. Efficiency gains were concentrated in normal studies, and the only randomised trial found lower mammography screen-reading workload with similar early screening performance. No study assessed patient outcomes, and 18 of 21 studies were retrospective. This evidence supports AI only as a supervised, complementary tool; improvements in diagnostic accuracy should not be equated with clinical benefit.

## Introduction and background

Diagnostic radiology is characterised by the continuous generation of large volumes of visual information requiring specialised interpretation, which can contribute to high workload and an increased risk of diagnostic error. Sustained growth in imaging volume, relative to radiology workforce capacity in many health systems, has renewed interest in tools that can support faster and more consistent image interpretation [1].

Artificial intelligence (AI) refers to computer systems that perform tasks that normally require human intelligence. Most current applications in diagnostic imaging rely on machine learning and, in particular, on deep learning, in which artificial neural networks, composed of successive layers of interconnected computational units, learn to recognise patterns directly from large sets of labelled images rather than from explicitly programmed rules. Convolutional neural networks, a deep learning architecture designed for image data, underpin most commercial tools for detecting findings such as fractures, pulmonary nodules or breast lesions. Radiology is the clinical specialty with the largest number of AI-enabled medical devices authorised by the US Food and Drug Administration [2], and these tools are increasingly used for detection, triage and quantification, and to support structured reporting [1].

Two distinctions are essential when interpreting this evidence. First, AI can be evaluated as a standalone system, whose output is compared directly with that of human readers, or as an assistive tool, in which readers using AI are compared with readers working without it; the first comparison addresses whether AI could replace part of human interpretation, and the second whether it improves the performance of the human reader. Second, task-specific image-analysis models, trained to detect or classify predefined findings, differ from generative AI, including large language models and multimodal models such as ChatGPT (OpenAI Group PBC, San Francisco, USA), which generate text in response to prompts and can be applied to image description, classification, or report generation. These technologies have different clinical applications, failure modes and performance profiles.

The quality of this evidence base, however, remains a concern. A systematic review of 535 deep-learning studies in radiology found that 98% were retrospective, only 31% used external validation and, among studies allowing direct comparison, performance decreased on average by 6% (range: 4% increase to 44% decrease) on external data [3]. A more recent meta-analysis of multi-reader multi-case studies in cancer imaging found that AI assistance increased pooled reader sensitivity from 0.67 to 0.79 and specificity from 0.82 to 0.87, but half of the studies raised concerns of bias and many used case-enriched image sets [4]. In addition, how AI outputs and their level of confidence are communicated to radiologists may influence how these tools are used and trusted in practice [5].

A number of primary studies have reported that AI systems can contribute to earlier detection of pathology, improved diagnostic accuracy and reduced interpretation time in specific radiological tasks. Given the heterogeneity of these reports, which span multiple imaging modalities, clinical settings and AI architectures, a systematic synthesis is needed to characterise the impact of AI on diagnostic accuracy and operational efficiency compared with standard human interpretation. Our objective was to describe this impact in diagnostic radiology, based on a systematically identified and source-verified body of primary evidence, analysing standalone and AI-assisted comparisons, and image-analysis and generative AI, separately.

## Review

Methodology

Study Design

A systematic review without meta-analysis of observational studies and clinical trials addressing the impact of AI tools on diagnostic accuracy and operational efficiency in diagnostic radiology, compared with standard human interpretation, was conducted and reported following the Preferred Reporting Items for Systematic Reviews and Meta-Analyses (PRISMA 2020) statement [6] and the methodological recommendations of the Cochrane Handbook for Systematic Reviews of Interventions [7]. The review question was structured using the population, intervention, comparison, outcome, time, and eligibility (PICOTE) framework shown in Table 1, and the eligibility criteria are shown in Table 2. The comparator criterion was met when AI was compared with human readers interpreting the same examinations, when readers' performance with and without AI assistance was compared, or when standalone AI was validated against a reference standard established by expert human readers. Studies reporting only prognostic outcomes, or only the textual quality of AI-generated reports, without a diagnostic-accuracy or operational-efficiency outcome were not eligible.

**Table 1:** Structured question — PICOTE CT: computed tomography; MRI: magnetic resonance imaging; CAD: computer-aided detection and diagnosis; AUC: area under the curve; PICOTE: population, intervention, comparison, outcome, time, and eligibility.

Item	Description
Population	Diagnostic-imaging studies (X-ray, CT, MRI, mammography, ultrasound) in patients treated in real clinical settings, interpreted by radiologists or other healthcare professionals.
Intervention	Use of AI tools (machine learning, deep learning, neural networks, CAD systems, generative/large language models) applied to image interpretation or report generation.
Comparison	Standalone AI compared with human readers (radiologists, other medical specialists or healthcare professionals) interpreting the same examinations, and/or human readers with versus without AI assistance; standalone AI validated against a reference standard established by expert human readers was also accepted.
Outcomes	Diagnostic accuracy (sensitivity, specificity, accuracy, AUC, Kappa) and/or operational efficiency (interpretation time, reporting time, workload, productivity, triage flow). Prognostic outcomes and textual report quality alone were not eligible outcomes.
Time	Studies published between 1 January 2019 and 11 April 2026 (date of the search), evaluating the diagnostic interpretation process in clinical practice.
Study design / Exclusion	Randomised controlled trials, prospective and retrospective observational studies (cohort, cross-sectional, case-control), and multi-reader validation studies comparing AI with human interpretation were eligible.

**Table 2:** Inclusion and exclusion criteria AI: artificial intelligence; AUC: area under the curve.

Type of criteria	Description
Inclusion	Original research articles with primary data, published in indexed, peer-reviewed journals, available in full text. Studies evaluating an AI tool applied to diagnostic radiology, published between 1 January 2019 and 11 April 2026, in English or Spanish, reporting at least one measure of diagnostic accuracy (sensitivity, specificity, accuracy, AUC or Kappa) or operational efficiency (interpretation time, reporting time, productivity, workload, or triage flow).
Exclusion	Records from databases outside the four searched, or duplicates across sources. Studies without full-text access. Studies evaluating model development only, without comparison with human readers or validation against an expert human reference standard. Studies reporting only prognostic outcomes or only the textual quality of AI-generated reports. Studies not directly evaluating AI in radiological image interpretation. Editorials, letters, conference abstracts without sufficient methodological detail, and non-systematic narrative reviews.

Search Strategy and Information Sources

A preliminary search of the International Prospective Register of Systematic Reviews (PROSPERO) was conducted to confirm the originality of the review question. This review was not prospectively registered in PROSPERO or another protocol registry; this is acknowledged as a limitation in the Discussion. A search strategy combining MeSH terms and free-text keywords (“Artificial Intelligence”, “Radiology”, “Radiologist”, “diagnostic accuracy”, “workflow efficiency”) was run in PubMed, ClinicalKey, ScienceDirect, and Google Scholar on 11 April 2026, covering studies published in English or Spanish between 1 January 2019 and the date of the search. The complete database-specific search strings and all applied filters are shown in Table 3. In PubMed, the article-type filter was restricted to clinical trials and randomised controlled trials; because this filter may have excluded eligible observational studies indexed in PubMed, this inconsistency with the eligibility criteria is acknowledged as a limitation. 

**Table 3:** Search strategy AI: artificial intelligence.

Database	Search string	Applied filters
PubMed	(“Artificial Intelligence” AND “Radiology” AND “Deep Learning” AND “Radiologist”) OR ((“efficacy” OR “effectiveness” OR “diagnostic accuracy”) AND “Radiology” AND “Artificial Intelligence”)	English and Spanish; human studies; full text available; clinical trials and randomised controlled trials
ClinicalKey	(“artificial intelligence in radiology” OR “deep learning radiology”) AND (“diagnostic accuracy” OR “clinical performance” OR “AI vs radiologists” OR “human vs artificial intelligence radiology”)	English and Spanish; clinical studies; AI vs. radiologist comparison
ScienceDirect	(“Artificial Intelligence” AND “Radiology”) AND (“diagnostic accuracy” OR “clinical performance” OR “AI vs radiologists”)	English and Spanish; last 8 years (2019–2026); narrative reviews, books and opinion pieces excluded
Google Scholar	(“artificial intelligence in radiology” OR “AI in radiology” OR “deep learning radiology”) AND (“AI vs radiologists” OR “comparison with radiologic interpretation”) AND (“workflow efficiency” OR “reading time radiologists”)	English and Spanish; first 200 results by relevance screened; full-text review

Google Scholar ranks results by relevance and does not allow complete export of large result sets; therefore, the first 200 records retrieved by the search string, sorted by relevance, were screened, in line with published recommendations to examine at least the first 200 to 300 results when Google Scholar is used in evidence reviews [8]. These records were screened by the same two reviewers using the same eligibility criteria as for the other sources. Table 2 shows the inclusion and exclusion criteria applied at both the screening and full-text stages.

Study Selection

Titles and abstracts of records identified through the search were screened against the eligibility criteria in Table 2, and the full texts of potentially eligible records were then retrieved and assessed in detail. Title and abstract screening and full-text eligibility assessment were performed independently and in duplicate by two reviewers; disagreements were resolved by discussion and consensus. When the full text of a potentially eligible record could not be accessed, retrieval was attempted by contacting the corresponding authors and by searching Google Scholar, ResearchGate, institutional repositories, and the journal website. Records whose full text could not be obtained after these attempts could not be assessed for eligibility and were not included; their potential impact is discussed among the limitations.

Data Extraction

Data were extracted independently and in duplicate by two reviewers using a standardised form, and discrepancies were resolved by discussion and consensus. Extracted items included author, year, country, study design, population and sample size, imaging modality, AI system and category (task-specific image-analysis AI or generative AI), type of comparison (standalone AI versus human readers, AI-assisted versus unassisted readers, or both), reference standard, and diagnostic-accuracy and operational-efficiency outcomes, with 95% confidence intervals (CIs) and p-values when reported in the original publication.

Source Verification

To ensure the accuracy of data extraction, each study proposed for inclusion was individually cross-checked against its original publication (full text obtained directly from the journal or publisher) to confirm authorship, study design, sample size, and the specific quantitative findings attributed to it. Studies whose reported characteristics or findings could not be confirmed against a matching original source were excluded rather than retained with unverified data. Records excluded during this process were counted within the full-text exclusions reported in the Results.

Data Synthesis

A quantitative meta-analysis was considered but judged inappropriate. Meta-analysis requires studies that are sufficiently similar in participants, index test, comparator, outcome, and methods for a pooled estimate to be meaningful [7]; for diagnostic accuracy, it also requires paired 2×2 data for the same target condition against a comparable reference standard, so that sensitivity and specificity can be pooled jointly. The included studies differed in imaging modality, clinical task and target condition, AI architecture and category, type of comparator (standalone versus assisted reading, and readers of different experience), reference standard, unit of analysis, case mix and disease prevalence (including case-enriched reader studies), and outcome metrics and thresholds, and few reported 2×2 data.

To examine whether a partial quantitative synthesis was possible, studies were grouped into subgroups sharing the same type of comparison, clinical task or outcome. For each subgroup, we recorded the number of studies, the outcome measures reported, the range of results, and the main sources of clinical and methodological heterogeneity. Pooling within a subgroup was considered feasible only if at least three studies reported the same outcome metric for the same target condition, with comparable reference standards and units of analysis and sufficient data for a bivariate model; otherwise, the subgroup was synthesised narratively. The synthesis was structured and reported in line with the Synthesis Without Meta-analysis (SWiM) guideline [9]. Results were stratified by AI category (task-specific image-analysis AI versus generative AI) and by type of comparison (standalone AI versus human readers, and AI-assisted versus unassisted readers, analysed separately); diagnostic-accuracy and operational-efficiency outcomes were reported separately, grouped by imaging modality, and interpreted in light of each study's risk of bias. Statistically significant differences were not assumed to be clinically relevant, and non-significant differences were not interpreted as evidence of equivalence or non-inferiority unless the original study had been designed to test them.

Risk of Bias and Quality Assessment

Quality Assessment of Diagnostic Accuracy Studies 2 (QUADAS-2) is the recommended tool for assessing the risk of bias of primary diagnostic accuracy studies [10], but it was designed for single-test accuracy studies and does not address the specific sources of bias of comparative designs, such as readers interpreting the same cases with and without AI, or of AI-based index tests. QUADAS-C extends QUADAS-2 to comparative accuracy studies [11], and an AI-specific extension, QUADAS-AI, has been proposed but was not available as a finalised tool when this review was conducted [12]. We therefore retained the four QUADAS-2 domains and their signalling questions, which apply to all included designs, and added AI-specific and comparative considerations derived from these extensions. Risk of bias was assessed independently and in duplicate by two reviewers, with disagreements resolved by consensus, across the four QUADAS-2 domains: patient selection, index test, reference standard, and flow and timing. Each domain was judged as low, high, or unclear risk using the QUADAS-2 signalling questions, with attention to AI-specific considerations: case enrichment or spectrum bias (patient selection); threshold or prompt selection and development and validation by the same team (index test); reference standards derived from the readers being evaluated or from dataset labels (reference standard); and, for comparative reader studies, whether readers interpreted the same cases with and without AI, the order of reading sessions, and the washout period (flow and timing). QUADAS-2 discourages summary quality scores; therefore, domain-level judgments are reported in full, and the following overall rating was used only to summarise and stratify the evidence, not to weight studies. Domain judgments were combined into a modified overall rating: low, when all four domains were at low risk; low-moderate, when one domain was unclear, and none was high; moderate, when one domain was high, or two or more domains were unclear; and moderate-high, when two or more domains were high.

Each study was also assigned a level of evidence adapted from the Oxford Centre for Evidence-Based Medicine hierarchy [13] (I: randomised controlled trial; II: well-designed prospective study; III: retrospective or cross-sectional study with a consistent reference standard; IV: case series) and a strength-of-recommendation grade (A: randomised evidence at low risk of bias; B: non-randomised evidence without the limitations listed for grade C; C: evidence limited by a small sample (fewer than 150 participants or examinations) or few target-condition cases (fewer than 20), case enrichment or highly selected samples, or a disclosed conflict of interest; D: high risk of bias). Because sample size and conflicts of interest are not QUADAS-2 domains, they were considered only in the grade. The grading criteria were applied uniformly to all studies. The level of evidence and the grade describe individual studies. The strength of the body of evidence for each subgroup was judged separately as high, moderate, low or very low, considering the domains used by Grading of Recommendations, Assessment, Development and Evaluation (GRADE) [14]: study design and risk of bias, consistency of findings, directness of the comparison and outcome to clinical practice, and precision. Evidence was rated high when consistent results came from at least two randomised trials at low risk of bias; moderate when results were consistent and included at least one level I or II study at low risk of bias; low when it came only from level III studies or when results were inconsistent; and very low when each task was addressed by a single level III study, or when the studies were mostly graded C and addressed different tasks. A formal GRADE assessment was not performed because no pooled estimates were produced.

Results

Study Selection

In the identification phase, 216 records were retrieved from the four databases on 11 April 2026; no records were identified from registers, and no records were removed by automation tools or for other reasons. After removal of 21 duplicates, 195 records were screened: seven were excluded on title and 92 on abstract, leaving 96 reports sought for retrieval. Twenty-three reports could not be retrieved despite the attempts described in the Methodology, and 73 were assessed in full text.

Fifty-two reports were excluded at the full-text stage: ineligible study design (n = 3), outcome of interest not reported or not verifiable against the original publication (n = 25), and absence of a human-interpretation comparator (n = 24). These counts include the six records excluded during source verification, namely one record that did not evaluate an AI tool in diagnostic radiology (counted as ineligible study design) and five records that could not be matched to a verifiable source publication (counted as outcome not reported or not verifiable), as well as one study reporting only the textual quality of AI-generated reports (outcome not reported) and one study reporting only prognostic outcomes without a human-reader comparison (no human-interpretation comparator). Twenty-one studies were included. The identification and selection process is summarised in the PRISMA 2020 flow diagram in Figure 1.

**Figure 1:**
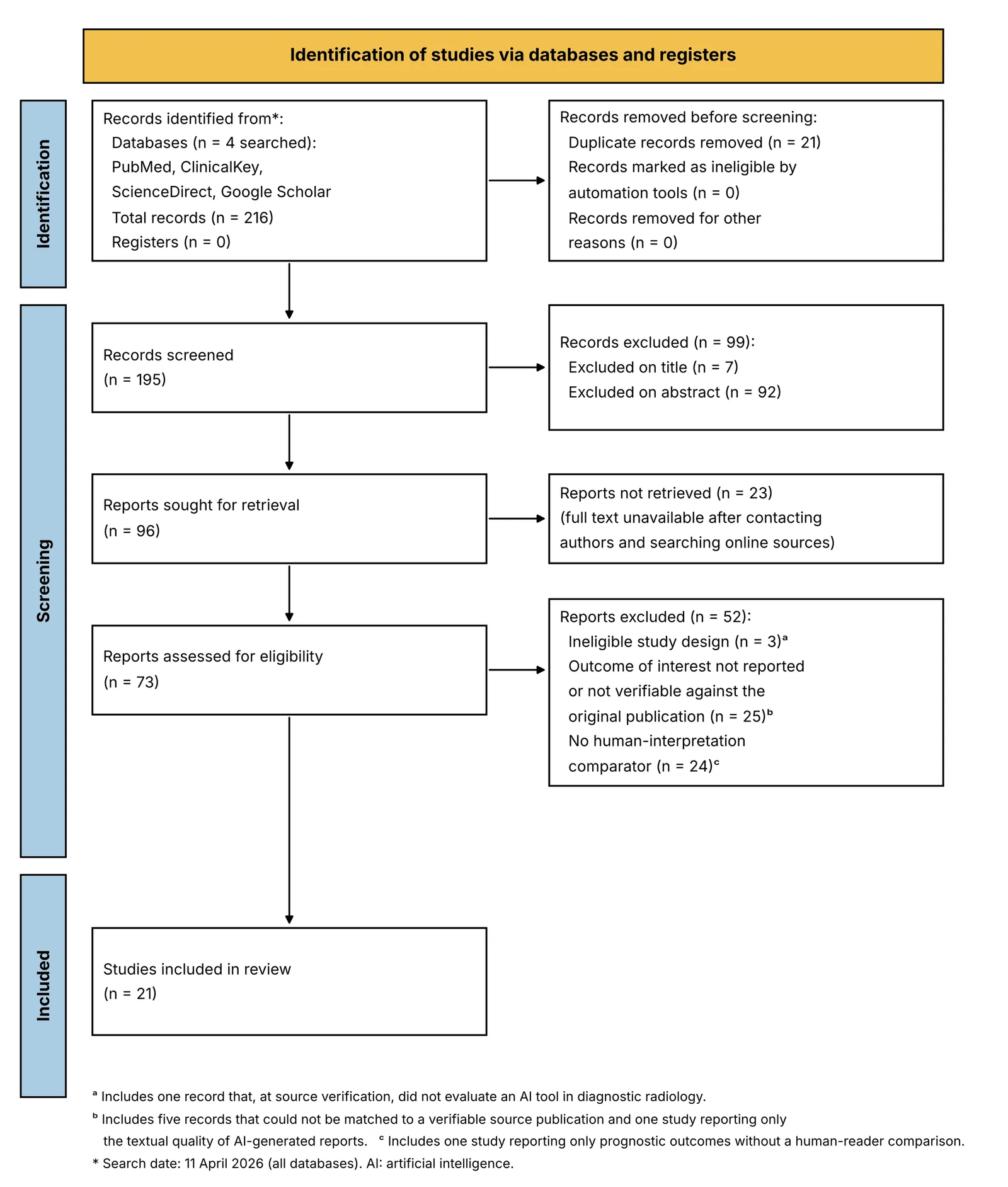
PRISMA 2020 flow diagram PRISMA 2020 flow diagram of study identification, screening and inclusion. Of 216 records identified from four databases, 195 were screened after removal of 21 duplicates; 96 reports were sought for retrieval, 23 could not be retrieved, 73 were assessed in full text, 52 were excluded and 21 studies were included.

Characteristics of the Included Studies

The 21 included studies [15-35] were published between 2019 and 2026 and comprised one randomised controlled trial, two prospective studies and 18 retrospective or cross-sectional studies (Table 4). Twelve studies evaluated radiography, three mammography, three MRI, two CT and one ultrasound. Eighteen evaluated task-specific image-analysis AI, and three evaluated generative AI models [24,26,28]. By type of comparison, 11 studies compared standalone AI with human readers [15-17,19,21,24,27,31,32,34,35], six compared AI-assisted with unassisted readers [18,20,23,28,30,33], and four reported both comparisons [22,25,26,29]; in two of the standalone studies, AI was compared with a reference standard established by expert readers rather than with a separate reader arm [17,19]. Four studies used case-enriched or highly selected samples, with a higher prevalence of disease than in routine practice [17,20-22].

**Table 4:** Summary of included studies AI: artificial intelligence; NPV: negative predictive value; COVID-19: coronavirus disease 2019; CT: computed tomography; AUC: area under the curve; RT-PCR: reverse transcription polymerase chain reaction; PPV: positive predictive value; CO-RADS: COVID-19 Reporting and Data System; MRI: magnetic resonance imaging; R-CNN: region-based convolutional neural network; CNN: convolutional neural network; MSK: musculoskeletal; GMIC (in Sydney-GMIC): Globally-aware Multiple Instance Classifier; κ: Cohen's kappa coefficient; MRMC: multi-reader multi-case; AUROC: area under the receiver operating characteristic curve; NS: not significant; DRCDS: Deep Learning-Radiologist Complementarity Decision System; ROT: Reflection of Thoughts; PROMIS: Prostate MR Imaging Study; nnU-Net: no-new-Net (self-configuring U-Net); COI: conflict of interest; DLAD-10: deep learning-based automatic detection algorithm for 10 common abnormalities; ResNet34: residual network with 34 layers; PI-RADS: Prostate Imaging Reporting and Data System; MASAI: Mammography Screening with Artificial Intelligence; CI: confidence interval; FPR: false-positive rate.

Author, year	Country	Study type	Population/sample	Modality	AI type	Comparison	Key findings
Silva-Afonso et al. [15]	Spain	Cross-sectional, diagnostic test evaluation study	346 chest X-rays + 261 bone X-rays, emergency department	Chest and bone X-ray	ChestView / BoneView	Standalone AI vs. human readers	ChestView: sensitivity 64.4% (vs. 49.2% emergency physicians, p=.018; vs. 83.9% expert radiologist, p<.001). BoneView: fracture sensitivity 87.5% (vs. 77.1% expert radiologist, p=.181; 87.5% emergency physicians), NPV 96.9%. Pneumothorax (4/346 cases): AI sensitivity 75.0%, NPV 99.7%. Prevalence: fracture 18.4% (48/261). Lower sensitivity for dislocation (60%) and joint effusion (25%).
Gallego-Díaz et al. [16]	Mexico	Observational, comparative, retrospective	192 patients with suspected COVID-19	CT	Automated quantification (Siemens)	Standalone AI vs. human readers	AI AUC vs. RT-PCR = 0.774; PPV 87.8%, NPV 58.8%. Human CO-RADS outperformed AI: sensitivity 91.8%, specificity 88.7%.
Wang et al. [17]	China	Retrospective, highly selected cohort	240 patients with rectal cancer	High-resolution MRI	Faster R-CNN	Standalone AI vs. expert reference standard	Accuracy 0.932, sensitivity 0.838, specificity 0.956, AUC 0.953; recognition time 0.2s.
Rudolph et al. [18]	Germany	Retrospective, multi-reader	563 chest X-rays; 3 radiologists, 3 residents, 3 non-radiologists	Chest X-ray	CNN-based AI system	AI-assisted vs. unassisted readers	In non-radiologists: pneumothorax AUC 0.846→0.974 (p<.001); nodule AUC 0.723→0.890 (p<.001). Smaller, non-significant gain in radiology residents.
Huhtanen et al. [19]	Finland	Retrospective, comparison of 2 algorithms	998 MSK radiographs (585 normal, 413 abnormal)	Conventional X-ray	BoneView and RBfracture	Standalone AI vs. expert reference standard	BoneView: sens. 0.893, spec. 0.885. RBfracture: sens. 0.872, spec. 0.892. No significant difference between them. Worse performance on subtle lesions.
Ueda et al. [20]	Japan	Retrospective, multi-institutional	312 chest X-rays (59 malignant); 18 readers	Chest X-ray	EIRL Chest X-ray	AI-assisted vs. unassisted readers	General physicians: sensitivity 0.47→0.60 with AI. Radiologists: 0.51→0.60. Greater benefit in less experienced readers.
Jiang et al. [21]	Australia	Retrospective, concordance analysis	540 cases (179 malignant, 361 normal); at least 73 radiologists	Screening mammography	Sydney-GMIC	Standalone AI vs. human readers	Agreement between AI and radiologists on case difficulty: Cohen's κ 0.88–0.92; lesion localisation agreement 83.2%. Intermediate/difficult cases: sensitivity AI 81.1% vs. radiologists 67.6% (p<.05); specificity 82.9% vs. 74.3% (p=.24).
Goh et al. [22]	Singapore	MRMC, enriched sample	500 women (250 with cancer, 250 without); 17 radiologists	Digital mammography	FxMammo	Both	AUROC AI 0.93 vs. consultants 0.90 (p=.21, NS). Greater gain in junior/senior residents with AI.
Zheng et al. [23]	China	Multicentre development and validation	994 patients	Contrast-enhanced CT	DRCDS	AI-assisted vs. unassisted readers	Improves liver fibrosis staging when combining AI with radiologist assessment; operational efficiency gain.
Castillejo et al. [24]	Spain	Cross-sectional, with control group	387 radiographs (129 with fracture)	Anteroposterior X-ray	Generative AI: ChatGPT-4.0	Standalone AI vs. human readers	Fracture detection: AI Kappa 0.98 (vs. 0.93 attendings, 0.848 residents). Schatzker classification: attendings outperformed AI (Kappa 0.616 vs. 0.612).
Crombé et al. [25]	France	Prospective, multicentre, real-life	969 patients (1049 radiograph series)	Limb/pelvis/shoulder X-ray	BoneView and SmartUrgence	Both	AI-assisted radiologists (accuracy 97.6%) outperformed emergency physicians (93%) and both stand-alone AI solutions (92.2–92.7%).
Jin et al. [26]	China	Retrospective, reader validation	103 cystic renal masses	Contrast-enhanced ultrasound	Generative AI: ChatGPT-4V (ROT prompt)	Both	Best-prompt AUC 0.688, comparable to junior reader (0.715) but inferior to senior reader (0.832, p=.019). Improved all three juniors to senior level.
Pooch et al. [27]	Netherlands	External, multi-cohort validation	573 prostate MRIs (PROMIS cohort)	Biparametric MRI	nnU-Net	Standalone AI vs. human readers	Model AUC fell on lower-quality images (0.63 vs. 0.71, p<.05); radiologists did not show this decline.
Hong et al.[28]	USA	Retrospective, multi-reader, public dataset	756 chest X-rays (PadChest)	Chest X-ray	Generative AI: KARA-CXR (Kakaobrain)	AI-assisted vs. unassisted readers	Reading time 34.2→19.8 s with AI-generated preliminary reports; sensitivity improved for several findings (e.g., widened mediastinal silhouette 84.3%→90.8%). Corresponding author discloses an employment relationship with the developer (COI).
Ziegner et al. [29]	Germany	Retrospective real-life + medicolegal subcohort	1672 radiographs (<18 years)	Bone X-ray	Commercial fracture software	Both	Sensitivity 92%, specificity 83%. Medicolegal fractures: 100% proximal tibia, 68% radial condyle. Residents with AI: sensitivity 83.7%→87.3%, specificity 90.7%→92.4%.
Shin et al. [30]	South Korea	Prospective, observational	18,680 chest X-rays; 11 radiologists	Chest X-ray	Commercial AI software	AI-assisted vs. unassisted readers	Total time: 13.3s (with AI) vs. 14.8s (without AI), p<.001. Normal cases: 10.8 vs. 13.1s. Cases with findings: no difference (18.6 vs. 18.4s, p=.452).
Nam et al. [31]	South Korea	Retrospective: development, external validation, simulated reading	146,717 training X-rays; simulated test with 6 radiologists	Chest X-ray	DLAD-10 (ResNet34)	Standalone AI vs. human readers	AUC 0.895–1.00. Detected more critical findings than radiologists (95.0% vs. 84.4%, p=.01). Reduced critical-case reporting time (640.5s vs. 3371.0s, p<.01).
Schelb et al. [32]	Germany	Retrospective, split-sample validation	312 men with suspected prostate cancer	Multiparametric MRI	U-Net	Standalone AI vs. human readers	U-Net sensitivity 92–96% vs. PI-RADS 88–96%, no statistical difference (p>.99).
Lång et al.[33]	Sweden	Randomised controlled, non-inferiority trial (MASAI trial)	80,033 women (40,003 AI / 40,030 double reading)	Screening mammography	Transpara v1.7.0	AI-assisted vs. unassisted readers	Cancer detection 6.1 (95% CI 5.4–6.9) vs. 5.1 (95% CI 4.4–5.8) per 1000; rate ratio 1.2 (95% CI 1.0–1.5), p=.052; above predefined safety limit (>3/1000). Screen-reading workload −44.3%. False-positive rate 1.5% in both arms. Interval cancer rate (primary endpoint) not reported in this analysis.
Pastor et al. [34]	France	Retrospective, single-centre	94 patients (71 fractures confirmed by CT)	Pelvis/hip/limb X-ray	Rayvolve (AZMed)	Standalone AI vs. human readers	Radiologist outperformed AI: sensitivity 92% vs. 82% (p=.045); specificity 88% vs. 69% (p=.016); accuracy 90% vs. 76% (p<.001).
Lee et al. [35]	South Korea	Retrospective: validation + screening	10,285 validation X-rays (10 cancers); 100,525 screening X-rays (47 cancers)	Chest X-ray	Commercial algorithm (Lunit)	Standalone AI vs. human readers	Validation: AUC 0.99, sensitivity 90% (AI) vs. 60% (radiologists), p=.25 (NS, wide CI); FPR 3.1% vs. 0.3% (p<.001). Screening: AUC 0.97.

Diagnostic Accuracy of Task-Specific Image-Analysis AI: Standalone AI Versus Human Readers

Radiography: Commercial fracture-detection algorithms achieved standalone sensitivities of 87.2-92% and specificities of 83-89% [15,19,29], with a fracture sensitivity numerically, but not significantly, higher than that of an expert radiologist in one emergency-department sample (87.5% vs. 77.1%, p=.181) and identical to that of emergency physicians (87.5%) [15]. Performance was lower for subtle lesions [19], medicolegally relevant fractures (sensitivity as low as 68% for radial condyle fractures) [29], dislocations (60%), and joint effusions (25%) [15]. In a real-life multicentre cohort, both standalone solutions (accuracy 92.2-92.7%) were outperformed by AI-assisted radiologists (97.6%) [25], and, with CT as the reference standard, a radiologist outperformed the AI solution in sensitivity (92% vs. 82%, p=.045), specificity (88% vs. 69%, p=.016) and accuracy (90% vs. 76%, p<.001) [34]. For chest radiographs, standalone AI was more sensitive than emergency physicians (64.4% vs. 49.2%, p=.018) but less sensitive than an expert radiologist (83.9%, p<.001), with a sensitivity of only 40.4% for consolidation [15]. A deep-learning algorithm detected more critical findings than radiologists (95.0% vs. 84.4%, p=.01) in a simulated reading study [31], whereas in a lung cancer validation set containing only 10 cancers, no statistically significant difference in sensitivity was detected (90% vs. 60%, p=.25) and the false-positive rate was higher with AI (3.1% vs. 0.3%, p<.001) [35].

Mammography: In a case-enriched multi-reader study (250 cancers and 250 normal cases), no statistically significant difference was detected between AI and consultant radiologists (area under the receiver operating characteristic curve (AUROC) 0.93 vs. 0.90, p=.21); this study was not designed to establish equivalence [22]. In a set of 540 screening mammograms (179 malignant) read by an AI model and at least 73 radiologists, cases that radiologists found difficult were also difficult for AI: agreement between AI and radiologists in assigning cases to difficulty categories was high (Cohen's κ 0.88-0.92), and they agreed on lesion localisation in 83.2% of cancer cases. In intermediate and difficult malignant cases, AI sensitivity was higher than that of radiologists (81.1% vs. 67.6%, p<.05), whereas the difference in specificity in intermediate and difficult normal cases was not statistically significant (82.9% vs. 74.3%, p=.24) [21].

CT and MRI: Radiologist-assigned COVID-19 Reporting and Data System (CO-RADS) scores outperformed an automated COVID-19 quantification tool on CT [16]. For prostate MRI, a U-Net model showed no statistically significant difference from clinical Prostate Imaging Reporting and Data System (PI-RADS) assessment (sensitivity 92-96% vs. 88-96%, p>.99) [32], and the area under the curve (AUC) of a deep-learning model fell on lower-quality examinations (0.63 vs. 0.71, p<.05), whereas radiologists' performance did not [27]. A Faster R-CNN model assessing the circumferential resection margin in rectal cancer achieved an accuracy of 0.932 against the expert reference standard in an explicitly highly selected cohort [17].

Diagnostic Accuracy of Task-Specific Image-Analysis AI: AI-Assisted Versus Unassisted Readers

AI assistance improved reader performance most consistently among non-radiologists and less experienced readers. In chest radiography, the AUC of non-radiologist physicians increased with AI from 0.846 to 0.974 for pneumothorax and from 0.723 to 0.890 for nodules (both p<.001), with smaller, non-significant gains among radiology residents; because the reference standard was derived from the radiologists evaluated, these gains should be interpreted with caution [18]. In a case-enriched lung cancer study, AI increased the sensitivity of general physicians from 0.47 to 0.60 and that of radiologists from 0.51 to 0.60, absolute increases of 13 and 9 percentage points, respectively [20]. Residents' patient-level fracture sensitivity increased from 83.7% to 87.3% and specificity from 90.7% to 92.4% with AI [29]; AI-assisted radiologists achieved the highest accuracy in a real-life fracture cohort [25]; junior and senior residents gained most from AI in mammography [22]; and combining AI with radiologist assessment improved liver fibrosis staging on CT [23]. In the MASAI randomised trial, AI-supported screening (AI-based triage to single or double reading) achieved a cancer detection rate of 6.1 (95% CI 5.4-6.9) per 1000 compared with 5.1 (95% CI 4.4-5.8) per 1000 with standard double reading (rate ratio 1.2, 95% CI 1.0-1.5, p=.052), above the predefined safety limit of more than 3 per 1000, with recall rates of 2.2% and 2.0% and a false-positive rate of 1.5% in both arms [33].

Diagnostic Performance of Generative AI Models

Generative models were evaluated separately because they are general-purpose systems, not trained for a specific detection task, whose output depends on prompting. ChatGPT-4.0 showed high agreement for tibial plateau fracture detection on radiographs (κ 0.98, vs. 0.93 for attending physicians and 0.848 for residents) but only moderate agreement for Schatzker classification, similar to that of attending physicians (κ 0.612 vs. 0.616) [24]. On contrast-enhanced ultrasound, the best-performing ChatGPT-4V prompt achieved an AUC of 0.688 for cystic renal masses, similar to a junior reader (0.715) but lower than a senior reader (0.832, p=.019), although its use raised junior readers' performance towards the senior level [26]. When radiologists used AI-generated preliminary chest radiograph reports, reading time fell from 34.2 to 19.8 seconds, and sensitivity improved for several findings (for example, from 84.3% to 90.8% for a widened mediastinal silhouette); the corresponding author disclosed an employment relationship with the developer of the evaluated model [28].

Operational Efficiency

Efficiency outcomes were reported in six studies. In a prospective study of 18,680 chest radiographs, AI reduced the total reading time per radiograph from 14.8 to 13.3 seconds (p<.001); the reduction was confined to normal (AI-negative) studies (13.1 vs. 10.8 seconds), whereas reading time for studies with findings was unchanged (18.4 vs. 18.6 seconds, p=.452) [30]. AI-supported mammography screening reduced the screen-reading workload by 44.3% [33], and AI-generated preliminary reports shortened chest radiograph reading time [28]. In a simulated reading study, AI-based prioritisation shortened the reporting time for critical cases (640.5 vs. 3371.0 seconds, p<.01) [31], an AI decision-support system improved the efficiency of liver fibrosis staging [23], and a rectal cancer model required 0.2 seconds per image, although without a human reading-time comparator [17].

Risk of Bias and Level of Evidence

Domain-level judgments are shown in Table 5. Patient selection was the domain most often at high risk, owing to case enrichment or highly selected samples in four studies [17,20-22]; the reference standard was at high risk in two studies, in which it was derived from the readers being evaluated [18] or from public-dataset labels [28], and the index test in one study, in which the best-performing prompt was selected on the test dataset [26]. Overall, three studies were at low risk of bias [25,30,33], six at low-moderate risk [15,19,27,29,34,35] and 12 at moderate risk. The randomised trial was the only study rated level I and grade A [33], two prospective studies were rated level II [25,30], and the remaining 18 were rated level III; eight studies were graded C because of case enrichment or selected samples [17,20-22], a small sample or few target-condition cases [26,34,35], or a disclosed conflict of interest [28].

**Table 5:** QUADAS-2-based risk-of-bias assessment and level of evidence of included studies Overall risk of bias: low, all domains at low risk; low–moderate, one domain unclear and none high; moderate, one domain high or two or more unclear; moderate–high, two or more domains high. Grade C: small sample (<150) or <20 target-condition cases, case enrichment or highly selected sample, or disclosed conflict of interest.

Study	Patient selection	Index test	Reference standard	Flow and timing	Overall risk of bias	Level of evidence	Grade	Main concern
Lång et al. [33]	Low	Low	Low	Low	Low	I	A	Randomised; large sample; interval cancer rate (primary endpoint) not reported in this analysis.
Shin et al. [30]	Low	Low	Low	Low	Low	II	B	Prospective, large sample; single centre; efficiency outcome only.
Crombé et al. [25]	Low	Low	Low	Low	Low	II	B	Prospective, multicentre, consecutive real-life patients.
Huhtanen et al. [19]	Unclear	Low	Low	Low	Low–moderate	III	B	Retrospective sample; reference standard by consensus of two specialists.
Silva-Afonso et al. [15]	Low	Low	Low	Unclear	Low–moderate	III	B	Random sample; reference by consensus of three radiologists, with additional imaging or clinical data when needed.
Pooch et al. [27]	Unclear	Low	Low	Low	Low–moderate	III	B	External validation in a different cohort, but retrospective.
Ziegner et al. [29]	Unclear	Low	Low	Low	Low–moderate	III	B	Retrospective real-life cohort plus non-consecutive medicolegal subcohort.
Pastor et al. [34]	Unclear	Low	Low	Low	Low–moderate	III	C	Small sample (94 patients); CT as reference standard.
Lee et al. [35]	Unclear	Low	Low	Low	Low–moderate	III	C	Only 10 cancers in the validation set; very wide CI.
Ueda et al. [20]	High	Low	Low	Low	Moderate	III	C	Case-enriched sample (59 malignant of 312).
Nam et al. [31]	Unclear	Unclear	Low	Low	Moderate	III	B	Retrospective; development and validation by the same team; simulated reading.
Schelb et al. [32]	Unclear	Unclear	Low	Low	Moderate	III	B	Retrospective, single centre; split-sample validation.
Rudolph et al. [18]	Unclear	Low	High	Low	Moderate	III	B	Reference standard derived from the radiologists being evaluated.
Gallego-Díaz et al. [16]	Unclear	Low	Unclear	Low	Moderate	III	B	Retrospective, single centre; RT-PCR as imperfect reference standard.
Castillejo et al. [24]	Unclear	Unclear	Low	Low	Moderate	III	B	Retrospective cross-sectional; prompt-dependent generative model.
Zheng et al. [23]	Unclear	Unclear	Low	Low	Moderate	III	B	Multicentre, but development and validation in the same study.
Jiang et al. [21]	High	Low	Low	Low	Moderate	III	C	Malignant-case enrichment; does not reflect screening prevalence.
Goh et al. [22]	High	Low	Low	Low	Moderate	III	C	Enriched 50/50 multi-reader design; may overestimate performance.
Wang et al. [17]	High	Unclear	Low	Low	Moderate	III	C	“Highly selected” cohort (acknowledged by the authors); internal validation.
Jin et al. [26]	Unclear	High	Low	Low	Moderate	III	C	Small sample (103 masses); best prompt selected on the test dataset.
Hong et al. [28]	Unclear	Low	High	Low	Moderate	III	C	Public-dataset labels as reference; declared conflict of interest.

Restricting attention to the nine studies at low or low-moderate risk of bias did not change the overall pattern: AI-assisted radiologists outperformed standalone AI in the real-life fracture cohort [25], standalone fracture algorithms had high sensitivity but lower performance for subtle lesions [15,19,29], a radiologist outperformed AI with CT as the reference standard [34], lower image quality degraded AI more than radiologists [27], efficiency gains were limited to normal studies [30], and AI-supported screening reduced workload with similar early screening performance [33]. Some of the results most favourable to standalone AI came from studies graded C because of case enrichment or few target-condition cases [22,35].

Subgroup Synthesis and Strength of Evidence

Eight subgroups of studies with similar comparisons, tasks or outcomes were identified (Table 6). None met the conditions for quantitative pooling. The largest subgroup, standalone fracture detection on radiographs, comprised five studies [15,19,25,29,34], but they differed in population (adults and children), algorithm, reference standard (expert-reader consensus or CT), unit of analysis and outcome metric, and one reported only accuracy; reported sensitivities ranged from 82% to 92% and specificities from 69% to 89%. In the other subgroups, studies used different outcome metrics for the same comparison, for example AUC, sensitivity or accuracy for AI-assisted reading [18,20,25,29] and reading time per examination, reporting time in a simulated workflow or screen-reading workload for efficiency [28,30,31,33], or addressed different target conditions. The strength of evidence was moderate for the direction of the effect of AI assistance on reader performance in radiography and for the reduction in screen-reading workload and reading time of normal studies, each supported by at least one level I or II study at low risk of bias [25,30,33]; low for standalone AI in radiography, mammography reader studies and prostate MRI; and very low for generative AI models and for single-study applications in CT and MRI.

**Table 6:** Subgroups of studies with similar comparisons and outcomes, feasibility of quantitative pooling and strength of evidence Quantitative pooling was considered feasible only if at least three studies reported the same outcome metric for the same target condition, with comparable reference standards and units of analysis. Strength of evidence: high, consistent results from at least two randomised trials at low risk of bias; moderate, consistent results including at least one level I or II study at low risk of bias; low, level III studies only or inconsistent results; very low, single level III study per task or studies mostly graded C addressing different tasks. AI: artificial intelligence; CT: computed tomography; AUC: area under the curve; AUROC: area under the receiver operating characteristic curve; κ: Cohen's kappa coefficient; RCT: randomised controlled trial; MRI: magnetic resonance imaging; PI-RADS: Prostate Imaging Reporting and Data System; COVID-19: coronavirus disease 2019.

Subgroup	Studies	Outcome measures	Range of reported results	Main sources of heterogeneity	Quantitative pooling	Strength of evidence
Standalone AI for fracture detection on radiographs	[15,19,25,29,34]	Sensitivity, specificity; accuracy only in one study	Sensitivity 82–92%; specificity 69–89%; accuracy 76–92.7%	Adults and children; different algorithms; reference standard (expert-reader consensus or CT); unit of analysis (patient or radiograph)	Not feasible	Low (level II–III; inconsistent, CT-referenced study favoured radiologist)
Standalone AI on chest radiographs	[15,31,35]	Sensitivity for different target conditions; AUC	Sensitivity 64.4% (all abnormalities), 95.0% (critical findings), 90% (lung cancer, 10 cases)	Different target conditions and comparators; simulated reading; few cancer cases	Not feasible	Low (level III)
AI-assisted vs. unassisted readers, radiography	[18,20,25,29]	Reader AUC, sensitivity, specificity, accuracy	AUC +0.13 to +0.17 (non-radiologists); sensitivity +3.6 to +13 percentage points; accuracy 97.6% with AI	Different metrics, target conditions, reader experience and reference standards	Not feasible	Moderate for direction of effect (consistent; includes one level II study at low risk)
Mammography: randomised screening trial and reader studies	[21,22,33]	Cancer detection rate, recall and workload (trial); AUROC, κ, sensitivity (reader studies)	Detection 6.1 vs. 5.1/1000; workload −44.3%; AUROC 0.93 vs. 0.90; κ 0.88–0.92	Randomised screening trial vs. case-enriched reader studies; different outcomes	Not feasible	Moderate for early screening performance and workload (one RCT); low for reader-study accuracy
Prostate MRI	[27,32]	Sensitivity vs. PI-RADS; AUC by image quality	Sensitivity 92–96% vs. 88–96%; AUC 0.63 vs. 0.71 by quality	Two studies; different outcomes and datasets	Not feasible	Low (level III)
Single-study applications in CT and MRI	[16,17,23]	AUC, staging performance, accuracy	AUC 0.774 (COVID-19 CT); accuracy 0.932 (rectal MRI)	Different target conditions; one study per task	Not feasible	Very low
Generative AI models	[24,26,28]	κ, AUC, reading time, sensitivity	κ 0.98 (fracture detection); AUC 0.688 (renal masses); reading time 34.2→19.8 s	Different tasks, modalities and models; prompt dependence; conflict of interest in one study	Not feasible	Very low (level III; two of three graded C)
Operational efficiency	[23,28,30,31,33]	Reading time per examination, reporting time, screen-reading workload	Reading time −1.5 s per radiograph overall (driven by normal studies) and −14.4 s with preliminary reports; critical-case reporting 640.5 vs. 3371.0 s (simulated); workload −44.3%	Different metrics; simulated vs. real workflow	Not feasible	Moderate for direction of effect (level I and II studies at low risk); magnitude and clinical relevance uncertain

Discussion

This review's findings are broadly consistent with an “augmented intelligence” model, in which AI functions as a support tool that can improve sensitivity and reduce specific components of workload without replacing the radiologist. Analysing the two types of comparison separately clarifies this pattern. Evidence that AI assistance improves human readers was relatively consistent, particularly for non-radiologists and less experienced readers [18,20,22,29], whereas evidence that standalone AI matches or exceeds human readers varied by task and was contradicted in a CT-referenced comparison [34] and in a real-life cohort in which AI-assisted radiologists outperformed standalone AI [25].

These findings are consistent with existing evidence. A meta-analysis of multi-reader multi-case studies in cancer imaging found that AI assistance increased pooled reader sensitivity from 0.67 to 0.79 and specificity from 0.82 to 0.87, with evidence that it helped less experienced readers close the gap with more experienced colleagues [4]; our review extends this observation beyond oncology to fracture and chest radiography. That meta-analysis also identified concerns of bias in half of the studies, frequent case enrichment, missing washout periods, and limited reporting of decision thresholds [4], limitations that we also observed. Conversely, the lack of consistent superiority of standalone AI in our review agrees with the observation that most radiology AI studies are retrospective and rarely externally validated, and that performance falls on external data [3].

Commercial fracture-detection algorithms, which are task-specific models trained on radiographs, achieved high standalone sensitivity [15,19,29], but their performance was lower for subtle and medicolegally relevant fractures [19,29] and below that of a radiologist when CT was the reference standard [34]. Generative models such as ChatGPT represent a different diagnostic approach: they are general-purpose systems that are not trained for a specific detection task and whose output depends on prompting. They approached junior-reader performance on narrow tasks but did not surpass specialists on more complex classification [24,26], and their results should not be combined with those of dedicated detection algorithms.

High negative predictive values (NPVs) were reported for pneumothorax (99.7%) and fracture (96.9%) in a single emergency-department sample of randomly selected radiographs [15]. Pneumothorax was rare in this sample (4 of 346 chest radiographs, 1.2%), and AI detected three of the four cases (sensitivity 75.0%), so the high NPV largely reflects the low prevalence; fractures were more frequent (48 of 261 bone radiographs, 18.4%), and emergency physicians achieved the same fracture sensitivity (87.5%) and a similar NPV (97.1%) [15]. Because NPV increases as prevalence decreases, these values apply to the prevalence of that sample and cannot be transferred to settings with a higher pretest probability, such as major trauma. They must also be read alongside the lower sensitivity reported for subtle fractures, dislocations and joint effusions [15,19,29]. An AI-negative result therefore cannot, on its own, exclude clinically significant findings, and it should complement rather than replace radiologist review.

Operational efficiency findings were more heterogeneous than a simple “AI saves time” narrative suggests. The statistically significant reduction in chest radiograph reading time corresponded to only 1.5 seconds per examination, or approximately 7.8 hours of reading time across 18,680 radiographs read by 11 radiologists over four months, and was confined to AI-negative studies [30]. Its practical relevance is therefore modest and may be greater in high-volume settings or at the triage stage, whereas validating AI-flagged findings may offset part of the gain. Larger reductions were reported for critical-case reporting time in a simulated workflow [31] and for reading time with AI-generated preliminary reports [28], but these come from simulated or reader-study settings and, in the latter, from a study with a disclosed conflict of interest.

Several issues limit the transfer of these results to routine practice. First, external validity: most included studies were retrospective; in three studies, the model was developed and validated by the same team [17,23,31], and AI performance declined with lower image quality [27]; in the wider radiology AI literature, performance decreased on average by 6%, and by up to 44%, when models were tested on data from other institutions [3]. Local validation before deployment and monitoring after deployment are therefore required. Second, false-positive results: AI was less specific than an expert radiologist for both chest radiograph abnormalities (89.9% vs. 96.1%, p=.010) and fractures on bone radiographs (87.8% vs. 95.8%, p=.003) in an emergency sample [15], and its false-positive rate for lung cancer was ten times that of radiologists (3.1% vs. 0.3%) [35]. In practice, false positives may lead to additional imaging, recalls, unnecessary referrals and extra validation work, which may offset efficiency gains [30]. Third, patient safety: no included study measured patient outcomes, such as missed diagnoses with clinical consequences, time to treatment or morbidity, and the only randomised trial reported early screening outcomes but not its primary interval-cancer endpoint [33]. Automation bias, in which readers accept incorrect AI outputs without cross-checking, is a recognised risk, and AI systems that report their level of confidence, for example by flagging doubtful fractures, may help radiologists decide when to re-examine an image [5]. Finally, routine use raises questions of interpretability, integration into the workflow, training of users and responsibility for errors [5], which were not addressed by the included studies.

AI performance also depended on image quality: a deep-learning model's AUC fell on lower-quality prostate MRI examinations, whereas radiologists' performance did not [27], reinforcing the need for image-quality control and human oversight when AI is deployed across variable acquisition conditions. In mammography screening, the only randomised evidence available [33] showed that, in a prespecified clinical safety analysis, AI-supported screening achieved a cancer detection rate above the predefined safety limit and similar to standard double reading, with similar recall and false-positive rates and a 44.3% lower screen-reading workload. This analysis did not report the trial's primary endpoint, the interval cancer rate, and its results are limited to these early screening outcomes; they do not establish non-inferiority or longer-term safety, and they derive from a single trial that should not be generalised to other modalities or tasks.

This review has limitations. First, the search was limited to four sources and to English and Spanish publications; Embase, Scopus and Web of Science were not searched, the PubMed article-type filter was inconsistent with the inclusion of observational studies and may have excluded eligible studies, and only the first 200 Google Scholar results were screened. Second, 23 reports could not be retrieved despite contacting authors and searching additional online sources; if these studies differed systematically from those retrieved, for example in reporting less favourable results, their exclusion may have introduced selection bias. Third, the review was not prospectively registered. Fourth, most included studies were retrospective, and several used case-enriched or highly selected samples [17,20-22], had small samples or few target-condition cases [26,34,35], or were affected by image quality [27]; enrichment with artificially balanced disease prevalence risks overestimating real-world performance. Fifth, 95% CIs could not be presented for every comparison, and heterogeneity precluded quantitative pooling. Sixth, the modified overall risk-of-bias rating, the study grades, and the subgroup strength-of-evidence ratings are not validated instruments, although the rules used are reported explicitly. Finally, one study involved a disclosed conflict of interest between the corresponding author and the technology developer [28].

## Conclusions

Across a heterogeneous evidence base in which 18 of 21 studies were retrospective and only one was a randomised trial, AI performed most consistently as a supervised second reader or triage tool in diagnostic radiology, and the strength of evidence was at best moderate. Three types of benefit should be distinguished. Improvements in diagnostic accuracy were shown mainly in retrospective and reader studies: AI assistance improved reader performance, particularly among less experienced readers, more consistently than standalone AI matched or exceeded human readers, and standalone performance was lower for subtle findings and lower-quality images. Benefits for clinical workflow were limited to reduced screen-reading workload in one randomised trial and modest reductions in reading time for normal studies. Benefits for patient outcomes were not assessed by any included study and therefore remain unproven. High negative predictive values depend on disease prevalence and coexist with reduced sensitivity for subtle abnormalities, so AI-negative results should not be used on their own to exclude clinically significant findings. Generative AI models remain below specialist performance on complex classification tasks. These findings support continued radiologist supervision and a complementary, rather than autonomous, role for AI, and prospective, externally validated studies that measure workflow and patient outcomes are needed before wider clinical adoption.
